# Do Seabirds Differ from Other Migrants in Their Travel Arrangements? On Route Strategies of Cory’s Shearwater during Its Trans-Equatorial Journey

**DOI:** 10.1371/journal.pone.0049376

**Published:** 2012-11-07

**Authors:** Maria P. Dias, José P. Granadeiro, Paulo Catry

**Affiliations:** 1 Eco-Ethology Research Unit, Instituto Universitário de Ciências Psicológicas, Sociais e da Vida, Lisboa, Portugal; 2 Museu Nacional História Natural e da Ciência, Universidade de Lisboa, Lisboa, Portugal; 3 Centro de Estudos do Ambiente e do Mar, Museu Nacional de História Natural e da Ciência, Lisboa, Portugal; Hawaii Pacific University, United States of America

## Abstract

Long-distance migrants have developed diverse strategies to deal with the challenges imposed by their annual journeys. These are relatively well studied in some avian groups, such as passerines, shorebirds and raptors. In contrast, few studies have addressed the migratory behaviour of pelagic birds in the light of current theories of optimal migration. Using a dataset of 100 complete migratory tracks gathered along four years, we performed a detailed study on the migratory strategy of a pelagic trans-equatorial migrant, the Cory’s shearwater *Calonectris diomedea*. We analysed daily routines, stopover ecology and travel speed, as well as the influence of the moon on several behavioural patterns. Cory’s shearwaters adopted a “fly-and-forage” strategy when migrating, similarly to what has been observed in some raptors. However, by flying by dynamic soaring, shearwaters attained high overall migration speeds, and were able to travel thousands of kilometres without making major stopovers and, apparently, without a noticeable pre-migratory fattening period. Other major findings of this study include the ability to adapt daily schedules when crossing major ecological barriers, and the constant adjustment of migration speed implying higher rates of travel in the pre-breeding movement, with a final sprint to the nesting colony. The present study also highlights a preference of Cory’s shearwaters for starting travel at twilight and documents a strong relationship between their migratory activity and the moon phase.

## Introduction

The long-distance migrations carried out by many animals every year entail considerable costs in terms of time, energy and, in some cases, predation risk [1], [2]. Bird species have developed diverse strategies to deal with the challenges faced during the migratory journey [2], [3] and both theoretical and empirical studies have investigated the extent to which species optimize the time or the energy expenditure along the trip [4]. Long-distance migrants, for instance, are expected to primarily minimize the time cost of migration [1], [3], [5], which can be achieved by stopping longer at the most favourable refuelling sites and accumulating enough body reserves to allow skipping low-quality potential stopovers [1]. Alternatively, or complementarily, birds can increase overall migration speed by foraging while they travel [6]. Nevertheless, because food is rarely plentiful along the entire migratory path, most long-distance migrants still need to regularly stop to refuel, or to accumulate fat reserves before departure [7].

Another major issue in migration ecology of birds is how individuals manage their daily routine in order to accomplish the need to fly, to forage and to rest [8]. For example, terrestrial long-distance migrants are generally nocturnal flyers, optimizing their daily schedule by flying during darkness and foraging during the day [9], [10]. For birds that fly while foraging or that make extensive search flights to find food it may pay to combine the activities of flying and foraging, adopting a “fly-and-forage” strategy [6]. Migrating by “fly-and-forage” may make the birds more prone to travel during daylight while searching for food (for diurnal foragers; [6], [11]). Nevertheless, such strategy is only possible when crossing potential foraging habitats [11]. When passing through ecological barriers, where foraging is not possible or is more difficult, birds are expected to change their daily routine [11], [12].

Several seabirds are long-distance migrants, including some of the most impressive travellers on Earth. Some notable examples are the Arctic tern *Sterna paradisea* and many shearwaters that travel tens of thousands of kilometres on their migratory journeys [13]–[17]. However, few studies have addressed the strategies used by these long-distance pelagic migrants in light of the current theories on optimal migration, probably due to the difficulty of studying the at-sea behaviour, a gap that recent technological developments may help to fill [18].

Seabirds are also among the fastest migrants, achieving overall travel speeds in the range of 400–1000 km per day [15], [19]. Like most other species, some seabirds are known to stopover during their migratory journeys [17], [20], [21], although the exact role and location of these pauses remains mostly unknown. The daily flight schedules of pelagic migrants are also poorly documented. Studies carried out during both breeding [22], [23] and wintering periods [24], [25] showed that flight activity of seabirds increase during moonlit nights, but none has examined the effect of the moon on their migratory behaviour.

Among seabird migrants, large or medium-sized petrels (Order Procellariiformes) are interesting for the fact that they profoundly differ in many aspects of their ecology and behaviour, when compared to other models often used for the study of bird migration, such as passerines and shorebirds [26]. In fact, petrels often fly by dynamic soaring, a technique that entails low energetic costs [27], but that is highly dependent on wind conditions to be effective [28]. When travelling with a favourable wind, the energy expenditure of a medium to large sized petrel can be almost as low as that achieved during incubation [28], [29]. Furthermore, by flying very close to the ocean surface, the costs involved in locating and taking prey while on the move are presumably lower than those expected for other long-distance migrants.

Here we present the results of a detailed study about the migratory strategy of a pelagic trans-equatorial migrant, the Cory’s shearwater *Calonectris diomedea*. Using a dataset with 100 migratory tracks we analyse the at-sea behaviour during long-distance journeys between the North and the South Atlantic [16], [20]. In the light of the theory of migration and the ecology of the species, we examine the following aspects of its general migration strategy: (1) do Cory’s shearwaters use a “fly-and-forage” technique, or do they just commute as quickly as possible, relying on previously accumulated reserves? (2) Do they maintain their daily routines along the entire migratory journey, or do they change their schedules along the route? (3) Are their daily routines affected by moonlight? (4) Do they migrate by steps separated by stopovers, or do they just move in one continuous journey? (5) How fast can they progress? Answers to such questions are not only interesting in the context of the natural history of pelagic birds, but they likely will motivate for further tests and refinements of several aspects of the theory of animal migration.

## Materials and Methods

### Ethics Statement

The deployment of geolocators (see below) did not take more than 10 minutes and on no occasion had visible deleterious effects on study animals. Carrying a geolocator did not affect the probability of return to breed during the following breeding season (see Methods S1). Previous studies have also shown that these devices do not negatively influence the breeding success of Cory’s shearwaters during both the previous or the following breeding events [30]. All work was approved by the relevant authorities (Instituto da Conservação da Natureza e da Biodiversidade and Serviço do Parque Natural da Madeira; research permits 107/2006, 116/2007, 107/2010/CAPT).

### Bird Tracking

We tracked the migration of 100 adult (48 males and 52 females) Cory’s shearwaters breeding at Selvagem Grande (30°02′ N; 15°52′ W) from 2006/07 to 2009/10, using leg-mounted geolocators. The geolocators were deployed at the end of each breeding season (August/September), and recovered at the beginning of the subsequent year (April–June).

The geolocators (mk7 model, developed by British Antarctic Survey and weighing 3.6 g, less than 0.5% of the weight of the birds, considering an average bird weight of 800 g [31]) recorded light intensity levels at 10 min intervals, sea surface temperature and saltwater immersion at 3 s resolution. Using light levels data we estimated the position of the birds twice a day, with an approximate accuracy of 186±114 km [32]. Light data were analysed using *TransEdit*, to check for integrity of light curves and to fit dawn and dusk times, and *Birdtrack* software, to estimate the latitude from day length and longitude from the time of local midday relative to Greenwich Mean Time. We assumed a sun elevation angle of −4.5 degrees, based on known positions obtained during ground-truthing of the loggers, carried out before and after deployment. Unrealistic positions (those resulting from interference of light curves at dawn or dusk, or around equinox periods) were removed from the analyses. The final part of the return migration of many Cory’s shearwaters coincides with spring equinox. Hence, the arrival dates at colony were estimated on the basis of longitude data, which are not affected by the proximity to the equinox, and indicated clear (eastward) variation when birds were approaching the nesting island [20].

### Data Analysis

We calculated several parameters related to the at-sea behaviour of Cory’s shearwaters, all derived from the saltwater immersion data (wet/dry) recorded by the geolocators: 1) percentage of time spent in flight; 2) average landing rate (number of landings.hour^−1^); 3) average foraging bout duration (in hours; see below); 4) average flight bout duration (in hours; see below) and 5) number of flight bouts. These parameters were calculated for each day during the non-breeding season (since the birds left the colony until their return the following breeding season), and also separately for the daylight and darkness periods of each day (local sunset and sunrise times were assessed by the light levels recorded by the geolocators). Additionally, we calculated 6) the percent of the flight period that occurred in darkness and 7) a night flight index, corresponding to the difference between the proportions of time spent in flight during darkness and during daylight, divided by the highest of these two values (the division by the highest value ensures a linear behaviour of the index in relation to the variation in values of diurnal and nocturnal activity); this index varies between −1 (flight activity restricted to daylight) and 1 (flight restricted to night), and a value of 0 corresponds to an allocation of the flight time during daylight and darkness proportional to the duration of each phase. We restricted these analyses to the individuals that migrated (i.e., that spent the non-breeding season away from the Canary Current; see [20]), resulting in a final dataset of 95 tracks.

We considered as a foraging bout a continuous period with several wet-dry transitions (a pattern that results from an episode of frequent landings and take-offs, considered a good predictor of foraging effort in seabirds; [29]; Fig. S1). The foraging bout interval criterion (*sensu* [33]; i.e., the minimum duration of an event - wet or dry - that was considered to split the foraging bouts) was estimated at 57 min, using a maximum likelihood approach proposed by [34]; see Methods S2). Although we believe that these episodes most probably reflect foraging events, we cannot rule out the possibility of a minor part of these also including other activities, such as social interactions. A flight bout was considered as any dry event that occurred during the inter-foraging-bout interval (i.e., a continuous period of flight that lasted at least 57 min.; Fig. S1).

We compared the daily activity patterns of Cory’s shearwaters among the several stages of the non-breeding period – outward migration (excluding stopovers), winter, return migration and stopovers. Although we were mainly focused on the activity patterns during the migratory phases, the behavioural data from the wintering stage (where birds are free from the parental care duties) were also included for comparative purposes. The existence of stopovers was evaluated using first-passage time (FPT) analysis [35], by locating areas of relatively intensive usage (i.e., longer FPTs) during the journey. We first identified the spatial scale at which stopovers may occur, by varying the range of radius from 200 to 1200 km. Based on the distribution of FTP at each scale, we first checked for the existence of stopovers whenever the FPT was longer than 4 days at a 200 km scale, 8 days at a 500 km scale and 20 days at a 1100 km scale. Given that all stopovers identified at larger scales were also identified at small ones, we defined as a stopover any position where FPT was longer than 4 days at a 200 km scale.

The effect of the lunar cycle was also considered in the comparisons between non-breeding phases; the fraction of the moon illuminated at midnight was obtained for each day from the United States Naval Meteorology and Oceanography Command (http://www.usno.navy.mil/USNO/astronomical-applications/data-services/frac-moon-ill). The data were grouped in three phases: new moon (less than 30% of moon illuminated), quarters (30–70% of illuminated moon) and full moon (more than 70% of illuminated moon). Each activity measure was first averaged by individual bird for each non-breeding stage and lunar phase; the values were then compared using a mixed effects generalised linear model, using the individuals as a random factor.

In order to check for the existence of a pre-migratory hyperphagia [7] consistent with presumed fat accumulation associated with the return migration, we analysed, for each individual, the daily activity patterns before leaving the wintering area (during a fortnight period preceding the departure date) and during mid-winter (on a fortnight period that occurred 28 days before the former, to avoid the potential effect of the moon). Values were then averaged per individual and per phase (mid- *vs* late winter) and compared using paired t-tests.

Analyses were carried out using R software [36] including the packages maptools, sp, proj4, lme4, diveMove and MASS. Distances (including those used to estimate the ground speed) were calculated as great circle distances. Except if otherwise stated, means are presented ± SE. Sample sizes differed among the analyses performed; a summary of the sample size used in each analysis is presented in Table S1.

## Results

### Migratory Routes and Stopovers

**Figure 1:**
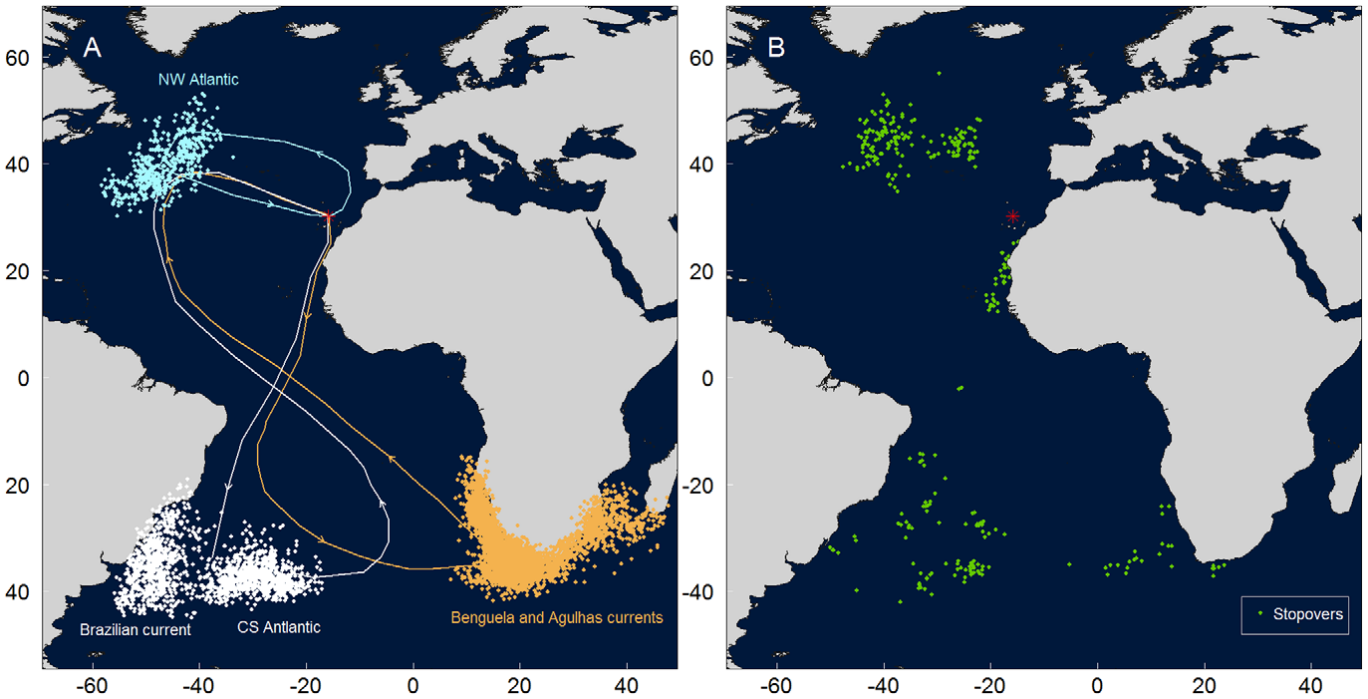
Examples of the migratory paths of Cory’s shearwaters to each wintering area (A) and stopovers locations (B). Dots represent wintering locations (panel A): orange – Benguela and Agulhas currents; white – Brazilian current and central South Atlantic; light blue – northwest Atlantic (the positions of the birds that stayed on Canary current are not shown). Green dots (panel B) represent stopovers locations. Red star indicates the colony location.

Cory’s shearwaters spent their wintering period in one of the following six areas: Benguela current (58%; n = 100), Agulhas current (14%), central South Atlantic (11%), Brazilian current (8%), Canary current (5%) and northwest Atlantic (4%). The main migratory pathways taken by Cory’s shearwaters to reach these areas are illustrated in Fig. 1A.

Stopovers were detected on 45 outward migratory journeys (47% of 95 tracks from birds that migrated); on 38 trips just one stopover was made, and on 7 trips the birds made two stopovers. Stopovers lasted, on average, 7.2±7.5 days (min.: 1; max.: 31), and were mainly located in known wintering areas used in other occasions or by other individuals (63%; n = 52 stopover locations), including the northwest Atlantic (a stopover that involve a detour of 5,000 km of additional distance travelled; Fig. 1B).

**Figure 2:**
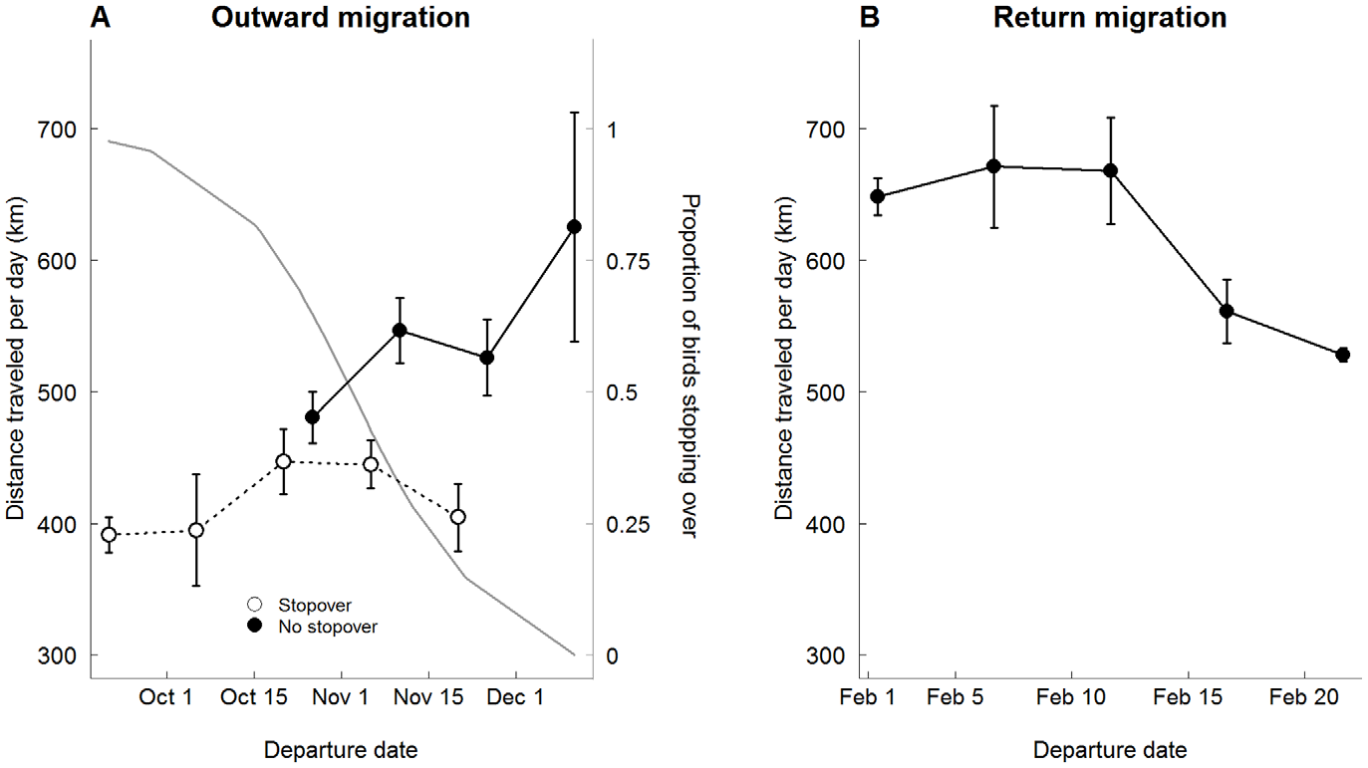
Relationship between departure date and mean (± SE) daily speed during the outward (A) and return (B) migrations. Open symbols (panel A) represent birds that stopped over (n = 33) and closed symbols birds that did not make stopovers (n = 30). Grey line represents the predicted probability of stopover occurrence considering the departure date from the colony (secondary y-axis; significant negative effect of departure date on the probability of stopping over during the outward migration; slope of the logistic regression = −0.045±0.02; *P = *0.03). None of the birds made stopovers during the return migration (panel B; n = 30).

Birds that left the colony later migrated faster (Pearson *r* = 0.29; *P* = 0.03; d.f. = 61), and made fewer stopovers than birds that left the colony earlier after the end of the breeding season (considering only the individuals that went to Benguela/Agulhas currents in order to avoid the possible influence of wintering location on migratory schedules and stopover probability); (Fig. 2A). Birds that did not make stopovers travelled considerably faster (535 km.day^−1^; comparing with 428 km.day^−1^ for birds that stopped: t = 5.31; *P*<0.001; d.f. = 52.12). The arriving time at the wintering areas did not differ between birds that stopped or not (10 and 5 of December, respectively; t = −1.58; *P* = 0.12; d.f. = 60.94). In the return migration, birds that departed earlier from the wintering areas travelled faster than those that departed later (Pearson *r* = −0.41; *P* = 0.03; d.f. = 28; Fig. 2B). None of the birds made stopovers during the return migration.

The mean overall migration speed (i.e., considering also the time spent on stopovers) was 429 km.day^−1^ and 644 km.day^−1^ during the outward and return migrations, respectively. The migration speeds achieved during the return migrations were ca. 200 km.day^−1^ faster than during the autumnal movements (paired t-test *t* = 5.73, d.f. = 23, *P*<0.001).

### Activity Patterns during Migration

When migrating, Cory’s shearwaters spent around 50% of the time flying (Table S2). Cory’s shearwaters migrated considerably more during daylight than during the night (Table 1). However, the major differences in relation to the activity patterns registered during the “stationary” stages (wintering and stopovers) were found on nocturnal flight activity: when actively travelling (i.e., outside the stopovers) Cory’s shearwaters more than doubled the time spent flying during darkness.

**Table 1:** Summary of the main activity patterns (means ± SE) of Cory’s shearwaters among the different stages of the non-breeding period.

	Migratory stages		Stationary stages	
	Outward	Return	Stopovers	Winter
Time spent in flight (%) in daylight	62.65±0.87	65.03±1.34	47.37±2.65	37.28±0.92
Time spent in flight (%) in darkness	37.11±1.20	42.32±1.94	24.17±2.3	15.86±0.95
Landing rate (per hour) in daylight	4.34±0.10	3.93±0.14	5.08±0.37	5.16±0.14
Landing rate (per hour) in darkness	3.99±0.29	2.68±0.19	6.1±0.95	4.85±0.35
Foraging bout duration (hours) in daylight	2.75±0.08	2.71±0.10	3.18±0.25	3.44±0.08
Foraging bout duration (hours) in darkness	2.03±0.07	1.97±0.18	2.04±0.20	2.28±0.07
Flight bout duration (hours) in daylight	1.74±0.03	1.66±0.04	1.52±0.10	1.65±0.03
Flight bout duration (hours) in darkness	2.06±0.04	2.1±0.06	1.83±0.09	1.91±0.05
Number of flight bouts (per day) in daylight	2.6±0.05	2.54±0.08	1.76±0.11	1.72±0.05
Number of flight bouts (per day) in darkness	1.4±0.05	1.77±0.08	0.95±0.10	0.52±0.03
Night flight index	−0.39±0.02	−0.33±0.03	−0.44±0.06	−0.55±0.02

During migration, birds landed less often, had shorter foraging bouts and longer and more frequent flight bouts (Table 1). The number of flight bouts in darkness was two to almost-four times higher during migration than during winter (Table S2). Cory’s shearwaters rarely flew continuously for more than four hours (95% of the flight bouts lasted less than this) and never more than 10 hours, with the mean duration of the flight bouts usually under two hours (Table 1).

**Figure 3:**
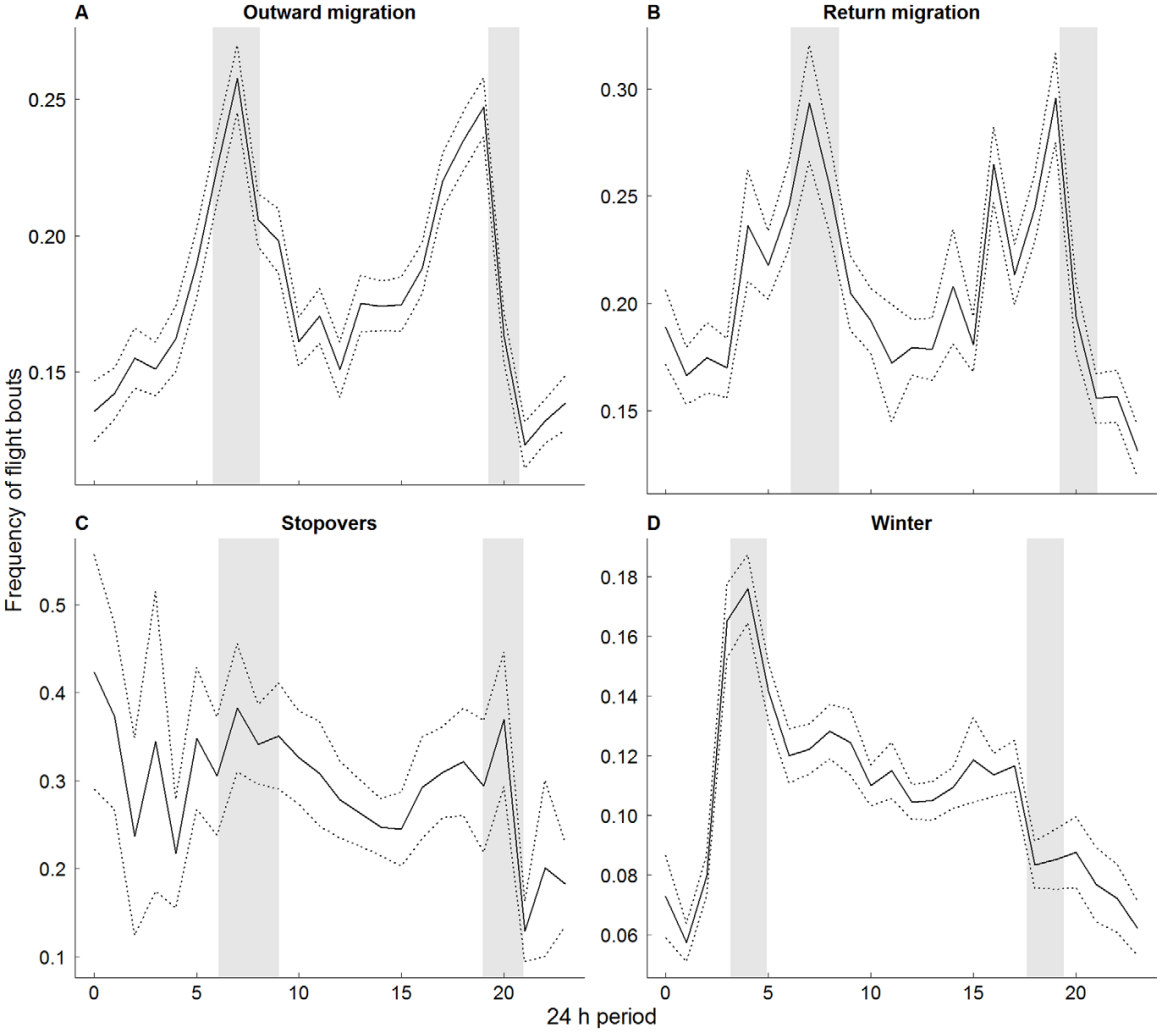
Frequency of individuals starting flight bouts during a 24 h period, in each stage of the non-breeding period. Solid line represents the mean and dashed line the SE. Grey areas correspond to sunrise and sunset intervals (mean ± SD).

When migrating, Cory’s shearwaters concentrated their flight activity around sunset and sunrise, a pattern that was not observed during winter (when there was a pronounced peak in flight initiation around sunrise only), or during the stopovers (when there were no pronounced peaks; Fig. 3).

### Moon Effect

**Figure 4:**
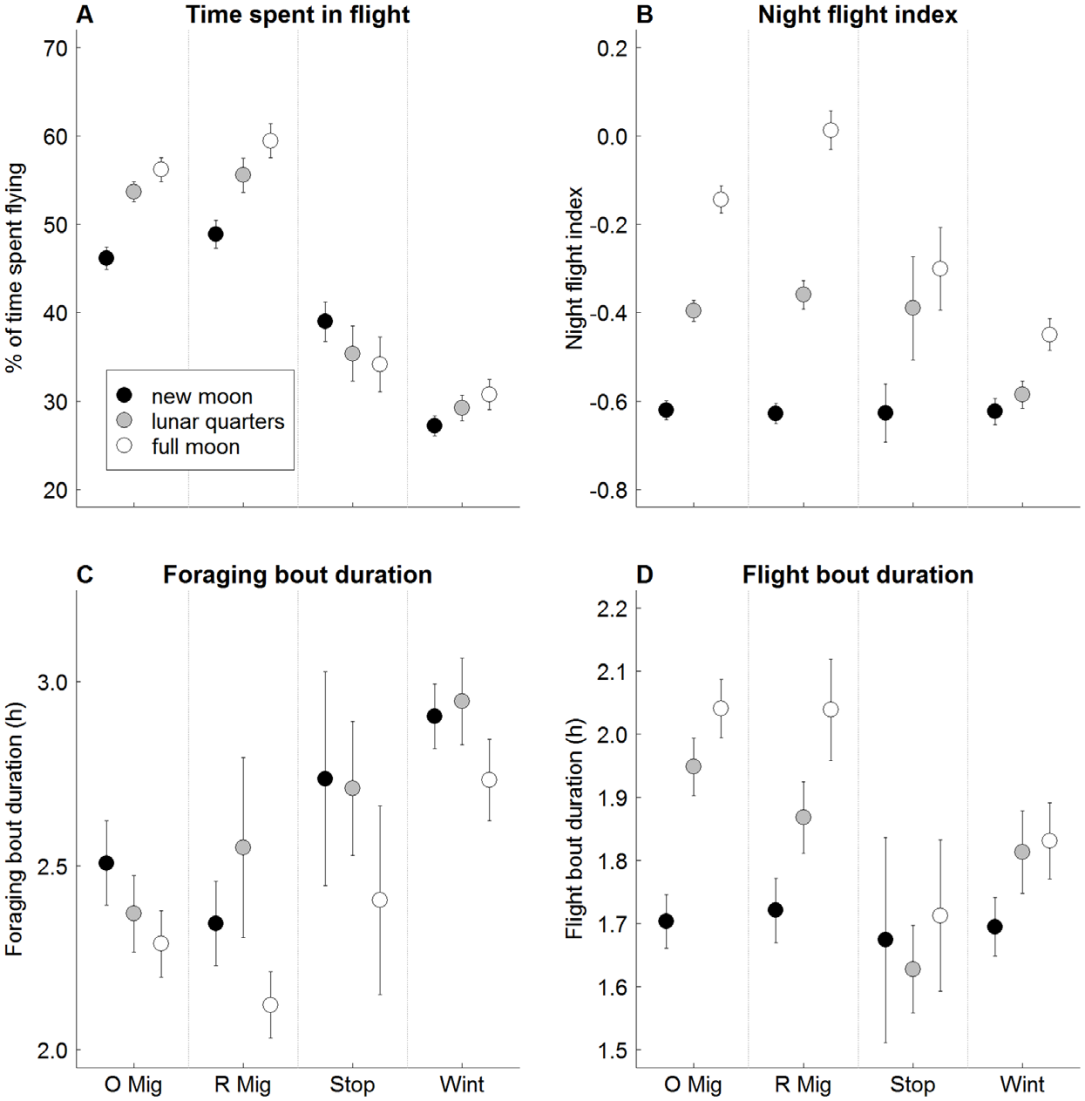
Comparison of some behavioural parameters among stages of the non-breeding period and lunar phases. O Mig – outward migration; R Mig – return migration; Stop: Stopovers; Wint – Wintering period; black circles – new moon; open circles – full moon; grey circles – lunar quarters (see methods). Means are presented ± SE.

The lunar phase had a strong influence on most activity parameters (Fig. 4), particularly (but not only) on those related with the night period (Table S2). The only exceptions were the landing rates, which were not affected by the moon.

On full moon days Cory’s shearwaters flew significantly more during darkness and less during daylight (Table S2), resulting in a much higher allocation of the flight time to the night period (Fig. 4B). The effects of the moon phase on the activity patterns were more pronounced during the migratory periods than during the winter and stopovers (Fig. 4).

The moon phase did not influence the timing of departure from the colony (comparison between the proportion of departure days and available days in each lunar phase: χ^2^ = 2.94; d.f. = 2; *P = *0.23) or from the wintering areas (χ^2^ = 3.24; d.f. = 2; *P = *0.20), neither was it associated with the date on which Cory’s shearwaters crossed the 10^th^ north parallel (χ^2^ = 0.49; d.f. = 2; *P = *0.78).

### Activity along the Migratory Path

**Figure 5:**
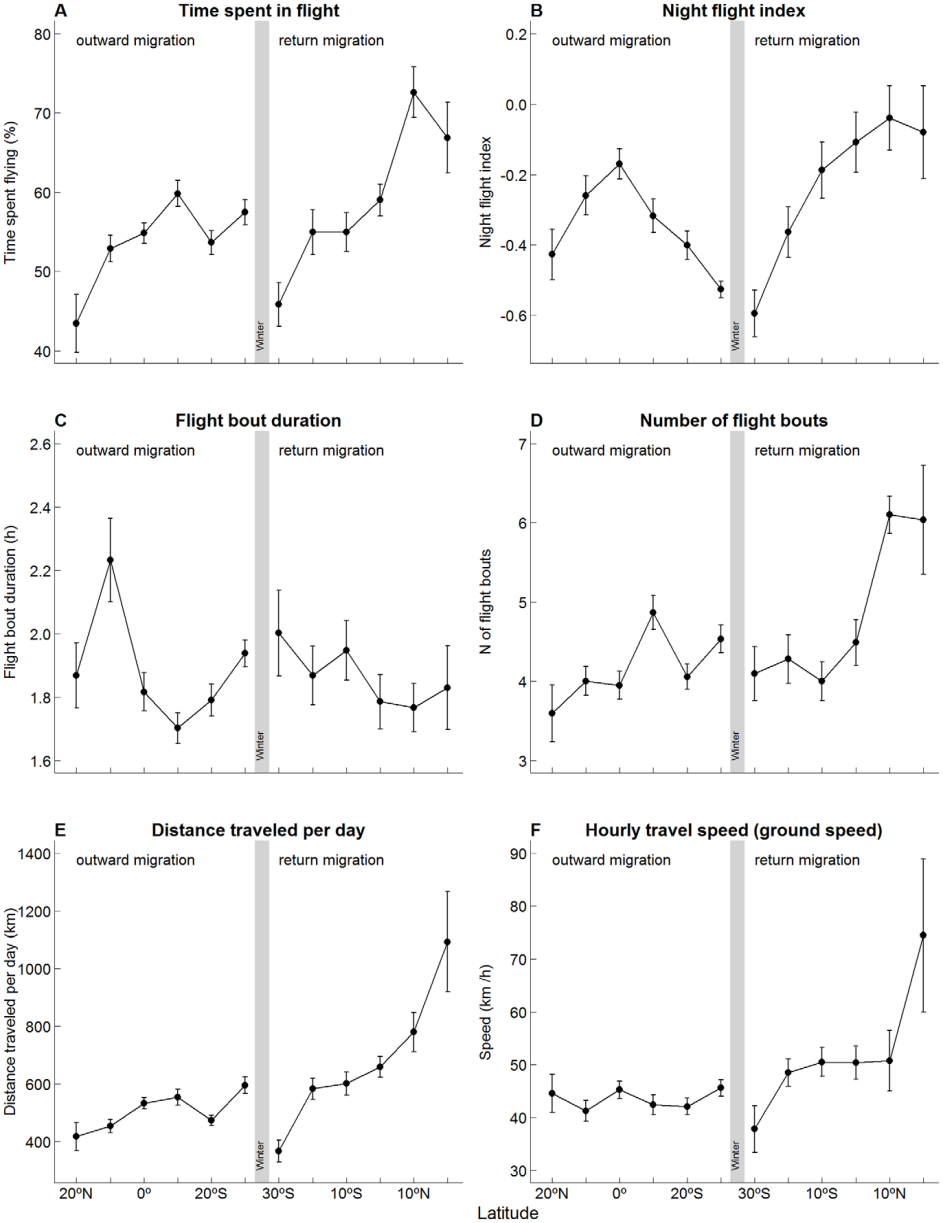
Variation in the flight activity patterns (means ± SE) along the latitude gradients crossed during the outward and return migrations. Note that latitude values (x-axis) varies southwards first (outward migration), and northwards after the grey bar (that represents the winter area), thus mirroring the temporal order at which the birds crossed each latitude.

**Figure 6:**
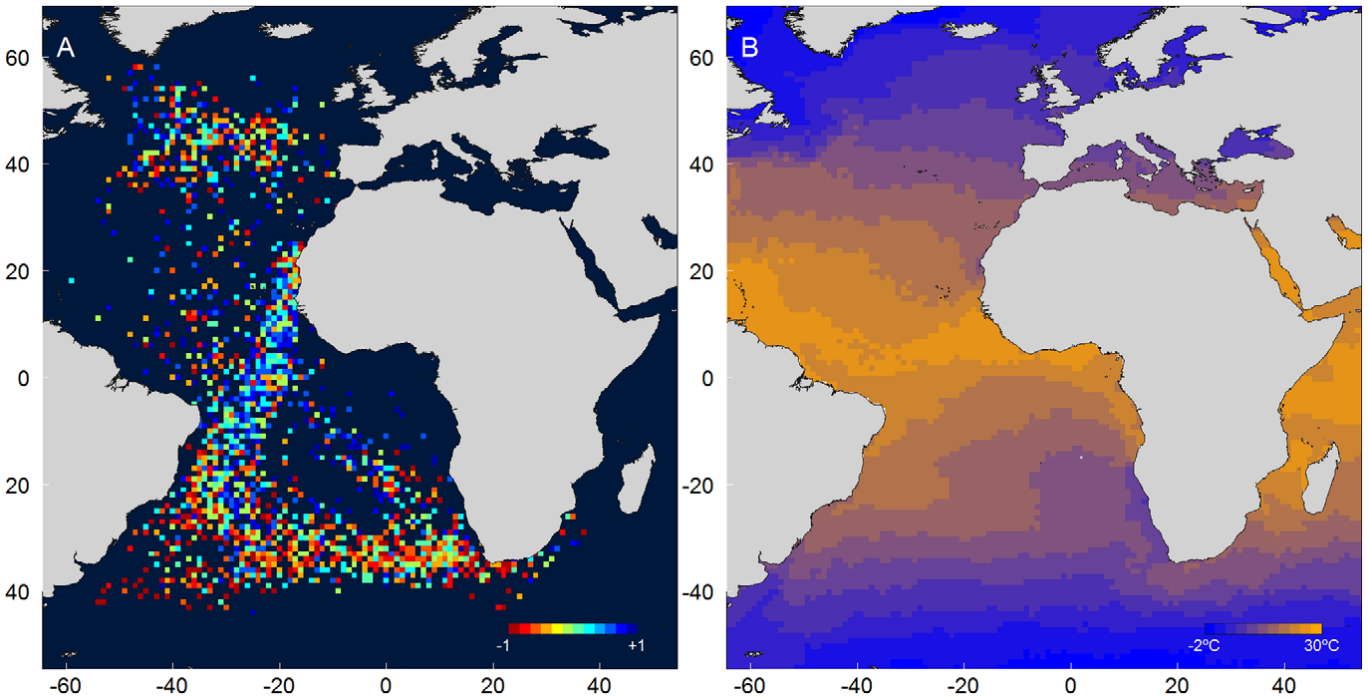
Variation of the night flight index (NFI) along the migratory routes (A) and sea surface temperature in the Atlantic Ocean (B). The NFI (panel A) reflects the amount of flight allocated to the night time, considering the relative duration of the night period (a value of 0 corresponds to a proportional allocation of the flight effort to the daylight and darkness; see methods); colours range from red (NFI = -1; i.e., flying exclusively during daylight) to blue (NFI = 1; i.e., flying exclusively during darkness). Colour gradient (panel B) reflects the sea surface temperature in the Atlantic Ocean during November 2007 and varies between blue (−2°C) and orange (30°C).

Activity patterns changed along the migratory journeys: Cory’s shearwaters spent more time flying when approaching the target areas (Fig. 5A). The night flight index peaked around the equator during the outward migration (Figs 5B and 6A), and during the final part of the return migration (Fig 5B). There were no obvious relationships between the duration of the flight bouts and latitude (apart from a peak around the 10^th^ north parallel; Fig. 5C). However, there was an increase in the number of flight bouts along the way (Fig. 5D), which resulted in an increase in the distance travelled per day, particularly obvious during the return migration (Fig. 5E). The flight speed (ground speed; measured considering only the time spent in flight) increased sharply when approaching the colony, but remained fairly constant during the outward migration (Fig. 5F).

### Pre-migratory Hyperphagia

In late winter, before the return migration, Cory’s shearwaters flew significantly more than they did during midwinter (Table 2). We did not find any other difference in Cory’s shearwaters behaviour between midwinter and pre-migration (Table 2).

**Table 2:** Comparison of the activity patterns (means ± SE) of Cory’s shearwaters between the pre-migratory phase of the wintering stage (a 15 days period before return migration: “-pre-migration”) and a period of equivalent duration 28 days before the former (“mid-winter”).

	Mid-winter	Pre-migration	Paired t-test
Time spent in flight (%)	23.43±1.28	27.58±1.41	*t* = 3.39; *P = *0.001; d.f. = 51
Landing rate (per hour)	4.43±0.23	4.51±0.25	*t* = 0.30; *P = *0.76; d.f. = 51
Foraging bout duration (hours)	2.78±0.13	2.72±.0.10	*t* = −0.41; *P = *0.68 d.f. = 45
Flight bout duration (hours)	1.64±0.05	1.66±0.05	*t* = 0.293; *P = *0.771; d.f. = 33
Number of flight bouts (per day)	1.93±0.12	2.08±0.14	*t* = 1.00; *P = *0.32; d.f. = 33
Night flight index	−0.67±0.03	−0.62±0.03	*t* = 1.61; *P = *0.11; d.f. = 51

## Discussion

To the best of our knowledge, this study represents the most detailed analysis available to date of the migratory behaviour of a seabird species. Our large data-set allowed investigating the at-sea activity patterns of many individuals with a very fine detail, and the high sampling frequency provided by the MK7 saltwater switch enabled an accurate estimation of several parameters of the behaviour of the birds, such as the landing rates (usually estimated indirectly; [23]). Furthermore, we adopted recent methodological developments for the identification of stopovers (using FPT; [35]) and of flying and foraging bouts [34].

### Overall Migratory Patterns

The individuals that were tracked followed the major migratory routes already known for the species [16], [20], [37], with the Benguela current representing the most important winter destination. Cory’s shearwaters travelled, on average, 430–644 km.day^−1^ (outward and return overall travel speed, respectively), a value considerably higher than that attained by most terrestrial long-distance migrants (which lies within the range of 20–200 km per day; reviewed by [3] and [10]), but in line with that observed in other marine migrants (such as long-tailed skuas *Stercorarius longicaudus* – 100–400 km.day^−1^; [38]; Arctic terns – 330–520 km.day^−1^; [13]; Sooty shearwaters *Puffinus griseus* – 500–900 km.day^−1^; [15], [39]). The high travel speeds attained by seabirds such as albatrosses and medium-sized petrels (that often reach 1000 km.day^−1^; [15], [17], [19]), can be explained by their wind-assisted flight method (dynamic soaring) that is much less energetically demanding than flapping flight, and allows birds to travel much more time without the need to rest or to fuel. In fact, the proportion of the time spent in flight by migrating Cory’s shearwaters (ca. 50%) is far higher than that registered for terrestrial migrants such as passerines and raptors (rarely higher than 20%, when considering all migratory period, i.e., stopovers included; reviewed by [10]).

Stopovers were only detected on a fraction of the journeys (ca. 40% of the outward, and none of the return), showing that Cory’s shearwaters are able to perform a 13,000 km trip (distance travelled between Selvagem and Benguela/Agulhas currents) without major breaks. Other seabirds, such as the Arctic terns [13], long-tailed skuas [38], South Polar skuas *Catharacta maccormicki* [21] and Manx shearwaters *Puffinus puffinus* [17] seem to stop much more often than Cory’s shearwaters, whereas the sooty shearwaters apparently stop less often [15]. The activity of birds at stopovers and so their exact role on the migratory ecology of seabirds is still poorly known. The few studies that so far examined the behaviour of shearwaters during this phase [17], [39] hypothesized that they may have an important refuelling function. Our results corroborate this: the activity patterns of Cory’s shearwaters during stopovers were, in general, similar to those observed on wintering areas, suggesting that during stopovers Cory’s shearwaters were indeed actively foraging.

Stopovers were mainly located in areas used for wintering by other Cory’s shearwater individuals, such as the Northwest Atlantic and the Central South Atlantic. The Northwest Atlantic region seems to be an important stopover area for several other seabird species too [13], [17], [21], [38]. About 10% of Cory’s shearwaters tracked in this study detoured more than 5,000 km from the main migratory pathway to spend between 15 and 31 days in this stopover. Similar important detours have been documented in other migrants such as the sharp-tailed sandpiper *Calidris acuminata* [40], and may result from birds targeting areas with particularly rich feeding conditions. Nevertheless, given that stopping over was mainly adopted by birds that left the colony earlier in the end of the breeding season, such detours can also be related with the lack of favourable wind conditions to cross the Intertropical Convergence Zone before mid October [41]. In fact, wind conditions are known to have an important role shaping migratory paths and phenology of shearwaters [41], [42]. The decision to stop or to carry on at any particular moment and site is also dependent on the body reserves of the migrant [43].

### The “Fly-and-Forage” Strategy of Cory’s Shearwaters

Our results show that migrating Cory’s shearwaters are mainly (but by no means exclusively) diurnal flyers. Most seabirds are more active during daylight, presumably due to a reduced foraging efficiency during the night [23], [44]. Nevertheless, many other long-distance migrants, such as several passerines, increase their nocturnal activity quite drastically during the migratory periods [10]. In fact, flying during the night period and feeding during the day can be the best strategy for diurnal foragers to minimize the total migration time [8]. This was not the case of Cory’s shearwaters, which maintained their essentially-diurnal flying schedule while migrating. Their flight activity was also characterized by several short flight bouts (approx. 2 h, on average), instead of the long flight bouts usual in some terrestrial long-distance migrants, like shorebirds and passerines crossing ecological barriers [45]. Even during active migration (i.e., outside stopovers), Cory’s shearwaters regularly switched between foraging and flying activities, particularly during daytime (Fig. S1). This strongly suggests that Cory’s shearwaters were adopting a “fly-and-forage” strategy, as reported for other migrants such as some birds of prey [6], [11], [12], and hypothesized to be widespread among birds that forage by using search flights [8].

Other strong indication that Cory’s shearwaters were adopting the “fly-and-forage” strategy comes from the large proportion of journeys accomplished without stopping over, including all the spring ones. If Cory’s shearwaters travelled without foraging, such journeys would only be possible if associated with an extensive pre-migratory fattening, for which there was little evidence: activity levels were not higher in the pre-migratory period than in the remaining wintering period. Interestingly, the sooty shearwaters, that are known to accumulate fat prior to return migration [14], seem also to adopt a “fly-and-forage” strategy (suggested from at-sea observations of foraging activity during their migration; [14]), but to a much lesser extent than Cory’s shearwaters do, as they spend up to 83% of the daylight period flying when actively commuting [39]. A possible explanation for this difference is that sooty shearwaters breed and winter at higher latitudes than Cory’s shearwaters, and typically forage on colder waters (averaging 4°–15°C; [46], comparing with ca. 20°C on Cory’s shearwaters; [31]), so it is possible that they are less adapted to forage in the (sub)tropical oceans they have to cross during their migratory journey.

For diurnal predators that perform extensive search flights to find their food the “fly-and-forage” strategy can help minimizing both the average time and energy spent on migration [8], despite the fact that there may be still some reduction in effective travel speed [6]. For example, if Cory’s shearwaters could keep migrating at full speed during the time devoted to foraging, they would potentially travel up to an additional 440 km each day (assuming that they generally did not keep the migratory heading during foraging bouts and considering the average time spent per day in foraging bouts – 10.4 h - and the average flight speed − 42 km/h). In reality, however, it is possible that the migratory heading is kept during part of the foraging bouts, and so the loss of ground covered is less than in the above estimate.

During migration, flight bouts were more often initiated around sunrise and sunset. This can be also related with the “fly-and-forage” strategy, given that the chances for the occurrence of multispecies feeding aggregations, on which shearwaters are common participants [14], [47], are probably higher during these periods, due to the increase of the feeding activity of subsurface marine predators such as dolphins and tuna [48], [49]. However, it is interesting to note that the flight activity peak around dusk was only observed during the migration periods, and not in the stopovers or in the wintering areas. This suggests that, like several other migrant birds [50], petrels choose to initiate travel bouts at a time when several potential orientation cues are available, namely the sun position, the horizon glow, the skylight polarisation pattern and the emerging star pattern [10].

### Moon Effect

Although Cory’s shearwaters are mainly diurnal migrants, the main differences in flight behaviour between migratory and stationary phases occurred during moonlit nights: on full moon days of active migration, Cory’s shearwaters flew almost as much during the darkness as during daylight. An increase in flight activity during moonlit nights had already been registered in several other seabirds outside the migration periods [22]–[25], but this effect is much more pronounced during travel. Phalan *et al.* [23] suggested that during full moon nights birds can more readily use visual cues to pursue their prey from the air. However, we did not detect any increase in the landing rate of Cory’s shearwaters on full moon nights, suggesting that the increase in flight effort on moonlit nights is not directly related with an increase in a foraging activity. An alternative explanation is that moonlight can simply increase the visibility during nocturnal flights [51]–[53]. Cory’s shearwaters fly at a close distance from the ocean surface, where the vertical gradient of wind speed (required for dynamic soaring) is at its maximum [54]. Darkness can simply hamper the manoeuvres used for dynamic soaring and consequently limit the use of this flight technique. It is also possible that the moon itself provides orientation cues, favouring travel when it is visible [51], [52].

### Differences between Outward and Return Migrations

Despite the general similarity in activity patterns between outward and return migrations, there were some noticeable differences. Cory’s shearwaters only made stopovers during the outward movement (and they also landed more often during this journey), resulting in a considerably faster return than outward migration. This pattern is in line with theoretical predictions and the general pattern found in many terrestrial long-distance migrants [4], [55], and is possibly due to the pressure to an early arrival to the colony to defend the nest [56]. Nevertheless, it is interesting to note that other trans-equatorial pelagic migrants, such as Manx shearwaters and South Polar skuas, stop during the outward migration as often as during the return migration [17], [21].

During the outward migration, birds that left the colony later in the season were the fastest, suggesting a pressure for an early arrival at the wintering areas (also indicated by the increase of the time spent in flight along the outward migration). Interestingly, we found the opposite pattern during the return migration – birds that left the wintering areas later in the season were the slowest. Nevertheless, the distances travelled per day during the return migration, even for these slower birds, were considerably higher than those attained during the outward migration. Cory’s shearwaters were particularly fast (600–1000 km.day^−1^) during the final part of the return migration, which was achieved by spending more time flying, but probably also by taking advantage of particularly good wind conditions (given the high values observed). This acceleration during the final phases of the return migration is predicted for birds that are competing for an early arrival to the nesting areas and that rely on resources obtained at the final destination for breeding, rather than transporting accumulated reserves into the area of reproduction [57].

### Equatorial Waters as an Ecological Barrier

The distribution of Cory’s shearwaters during the non-breeding season is associated with major upwelling systems and other productive areas of the Atlantic [16], avoiding the warm, oligotrophic sectors mainly located around the equator (Fig. 6B). However, when commuting from breeding to wintering areas and vice-versa, Cory’s shearwaters need to cross this latter area, which potentially acts as an ecological barrier [2]. The lower productivity of this oceanic sector in autumn [58] can directly affect the foraging efficiency of Cory’s shearwaters, forcing adjustments on their travel routines.

Other migrants have been shown to increase the number of travelling hours per day when crossing barriers such as the Sahara desert [11], [12]. A similar increase in Cory’s shearwaters overall flight activity was not obvious from our data, but the day *versus* night allocation of flight effort was dramatically different around the equator, a pattern that was particularly obvious when “mapping” their behaviour (Fig. 6A,). When travelling through the equatorial warm waters, Cory’s shearwaters increased the proportion of time flying in darkness, particularly during the autumnal migration. It is important to note here that we did not find any significant relationship between the timing of migration and the moon phase. The above finding corroborates the recent theoretical models of optimal migration [8] that predict that a strategy of combined diurnal and nocturnal migration would minimize both time and energy costs of long-distance migrants crossing ecological barriers. According to this author, another situation that would favour flying both during daylight and darkness would be particularly favourable wind conditions, as possibly happen with Cory’s shearwaters at the final stages of return migration (see above).

### Conclusions

The present study shows that Cory’s shearwaters uses mainly a “fly-and-forage” strategy to complete its annual journey of tens of thousands of kilometres between breeding and wintering areas. This strategy is quite similar to what is observed in other long-distance migrants that forage from the air, such as the ospreys *Pandion haliaetus* and Eurasian hobbies *Falco subbuteo* [6], [8], [11]. All these species also share the ability to adapt their daily schedules to the conditions found en route [11], [12], particularly when crossing major ecological barriers. The major difference in the travelling behaviour between Cory’s shearwaters and other long-distance migrants (as raptors, shorebirds and passerines) is the remarkable capacity of this species to travel thousands of kilometres without stopping over and possibly without extensive previous fattening. To a large extent the economy of this migratory strategy builds on the ability of Cory’s shearwaters to extensively use dynamic soaring, which allows high overall migration speeds.

## Supporting Information

Figure S1 **Example of a daily activity pattern of a Cory’s shearwater during its outward migration.**
(PDF)

Table S1 **Sample sizes for the analyses.**
(PDF)

Table S2 **Comparison of activity patterns (means ± SD) of Cory’s shearwaters among the several stages of the non-breeding period and moon phases.**
(PDF)

Methods S1 **Effect of the loggers on the probability of return to breed on the following breeding season.**
(PDF)

Methods S2 **Identification of the foraging bouts.**
(PDF)
