# Supplementary material for: Do Seabirds Differ from Other Migrants in Their Travel Arrangements? On Route Strategies of Cory’s Shearwater during Its Trans-Equatorial Journey
Source: PLoS One. 2012 Nov 7;7(11):e49376. doi: 10.1371/journal.pone.0049376 (PMC3492286; doi:10.1371/journal.pone.0049376)

Supporting information to manuscript: *Do Seabirds Differ from Other Migrants in their Travel Arrangements? On Route Strategies of Cory's Shearwater during its Trans-Equatorial Journey* by Maria P. Dias, José P. Granadeiro & Paulo Catry

Figure S1. Example of a daily activity pattern of a Cory's shearwater during its outward migration. Dashed vertical lines represent sunset and sunrise. Horizontal bars represent foraging bouts (dark grey) and flight bouts (light grey).

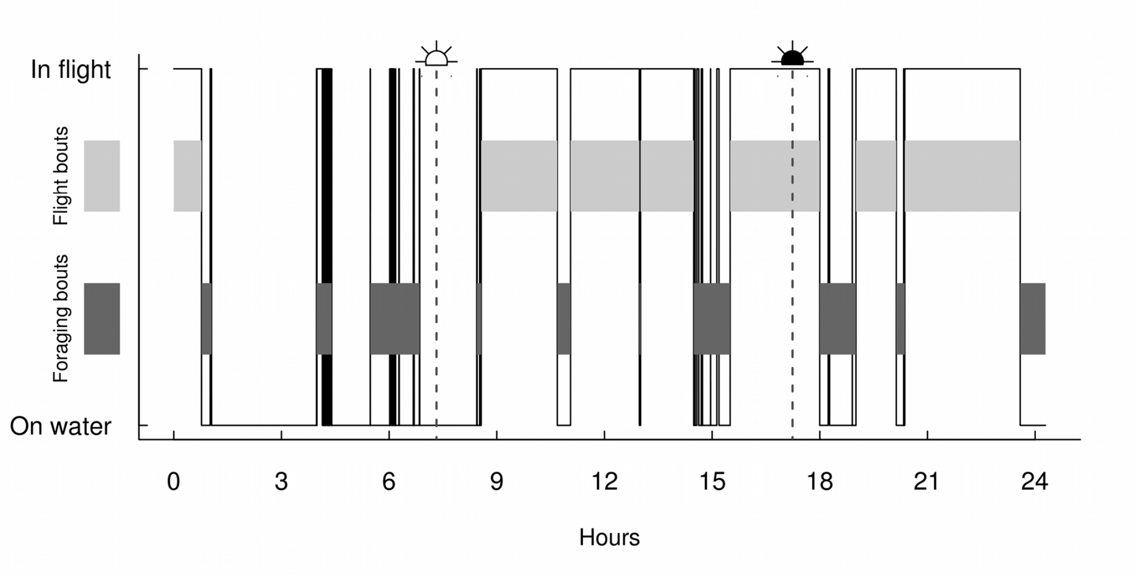

Supplement: Figure S1 — Example of a daily activity pattern of a Cory’s shearwater during its outward migration. (PDF) [file pone.0049376.s001.pdf]
