# Supplementary material for: Do Seabirds Differ from Other Migrants in Their Travel Arrangements? On Route Strategies of Cory’s Shearwater during Its Trans-Equatorial Journey
Source: PLoS One. 2012 Nov 7;7(11):e49376. doi: 10.1371/journal.pone.0049376 (PMC3492286; doi:10.1371/journal.pone.0049376)
Supplement: Table S1 — Sample sizes for the analyses. (PDF) [file pone.0049376.s002.pdf]

Supporting information to manuscript: *Do Seabirds Differ from Other Migrants in their Travel Arrangements? On Route Strategies of Cory's Shearwater during its Trans-Equatorial Journey* by Maria P. Dias, José P. Granadeiro & Paulo Catry

Table S1. **Sample sizes for the analyses**

Analysis	Number of individuals	Notes
Overall proportion of birds wintering in each area	100	All individuals tracked
Occurrence of stopovers	95	Birds that migrated [A]
Relationship between departure date, stopover occurrence and speed of outward migration	63	Good quality data [B] on outward journeys to Benguela/Agulhas currents [C]
Relationship between departure date and speed of return migration	30	Good quality data [B] on return journeys from Benguela/Agulhas currents
Comparisons of speeds between outward and return migrations	24	Good quality data [B] for both journeys (to and from Benguela/Agulhas currents)
Activity patterns during migration	95	Birds that migrated [A]
Moon effects	95	Birds that migrated [A]
Activity along the migratory path	95	Birds that migrated [A]
Pre-migratory hyperphagia	95	Birds that migrated [A D]

[A] – i.e., that spent the non-breeding season away from the Canary Current;
[B] – in what concerns geolocation data;
[C] – degrees of freedom may vary among t-tests due to the Welch correction;
[D] – degrees of freedom may vary due to the removal of bad quality activity data during the winter (tagged as "suspect" after decompression);
