# Supplementary material for: Do Seabirds Differ from Other Migrants in Their Travel Arrangements? On Route Strategies of Cory’s Shearwater during Its Trans-Equatorial Journey
Source: PLoS One. 2012 Nov 7;7(11):e49376. doi: 10.1371/journal.pone.0049376 (PMC3492286; doi:10.1371/journal.pone.0049376)
Supplement: Table S2 — Comparison of activity patterns (means ± SD) of Cory’s shearwaters among the several stages of the non-breeding period and moon phases. (PDF) [file pone.0049376.s003.pdf]

Supporting information to manuscript: *Do Seabirds Differ from Other Migrants in their Travel Arrangements? On Route Strategies of Cory's Shearwater during its Trans-Equatorial Journey* by Maria P. Dias, José P. Granadeiro & Paulo Catry

Table S2. Comparison of activity patterns (means ± SD) of Cory's shearwaters among the several stages of the non-breeding period and moon phases.

	Moon phase	Outward Migration	Return Migration	Stopovers	Winter	GLM
Time spent in flight (%)	New moon	46.14±10.74	48.89±9.26	38.98±7.40	27.21±8.30	Stage***
	Quarters	53.68±9.88	55.57±11.62	35.38±9.92	29.22±10.49	Moon***
	Full moon	56.2±11.47	59.45±11.30	34.14±10.78	30.72±12.37	Interaction**
Time spent in flight in daylight (%)	New moon	63.46±11.86	69.42±13.18	55.94±14.56	36.76±10.31	Stage***
	Quarters	65.01±13.13	67.48±12.07	46.63±16.09	37.71±11.33	Moon***
	Full moon	59.23±13.27	58.04±13.35	40.13±11.74	37.37±13.03	Interaction**
Time spent in flight in darkness (%)	New moon	22.98±12.84	24.94±9.43	19.15±11.54	12.12±8.51	Stage***
	Quarters	37.47±12.26	41.31±15.14	25.47±14.02	15.28±11.29	Moon***
	Full moon	50.84±16.57	60.78±15.20	27.68±13.59	20.08±14.27	Interaction***
% of the flight that occurs in darkness	New moon	21.01±9.14	21.85±6.01	23.82±12.85	17.21±8.64	Stage***
	Quarters	30.12±9.85	33.29±8.35	34.89±13.36	19.26±10.73	Moon***
	Full moon	38.68±1.67	46.13±9.57	35.39±13.79	24.47±11.78	Interaction***
Night flight index	New moon	-0.62±0.18	-0.63±0.14	-0.63±0.22	-0.62±0.22	Stage***
	Quarters	-0.40±0.21	-0.36±0.19	-0.39±0.37	-0.59±0.22	Moon***
	Full moon	-0.14±0.26	0.01±0.26	-0.30±0.32	-0.45±0.27	Interaction***
Landing rate (per hour)	New moon	4.34±2.50	3.44±1.03	6.13±3.36	5±1.74	Stage***
	Quarters	4.21±2.35	3.29±1.31	5.82±3.49	4.97±1.55	Moon $^{n.s.}$
	Full moon	4.28±2.33	3.37±1.08	5.31±2.83	5.24±1.99	Interaction $^{n.s.}$
Landing rate (per hour) in daylight	New moon	4.4±1.50	3.82±1.27	5.56±2.44	5.17±1.78	Stage***
	Quarters	4.25±1.38	3.73±1.38	5.3±2.22	5.18±1.85	Moon $^{n.s.}$
	Full moon	4.38±1.75	4.24±1.55	4.47±1.71	5.11±1.83	Interaction $^{n.s.}$
Landing rate (per hour) in darkness	New moon	4.12±4.67	2.95±1.81	6.09±5.90	4.76±4.47	Stage***
	Quarters	3.98±4.27	2.77±2.3	5.92±5.60	4.44±3.68	Moon $^{n.s.}$
	Full moon	3.88±40	2.31±1.59	6.26±5.49	5.34±4.83	Interaction $^{n.s.}$
Foraging bout duration (hours)	New moon	2.51±0.96	2.34±0.67	2.74±0.96	2.91±0.64	Stage***
	Quarters	2.37±0.92	2.55±1.47	2.71±0.58	2.95±0.85	Moon*
	Full moon	2.29±0.76	2.12±0.53	2.41±0.89	2.73±0.81	Interaction $^{n.s.}$
Foraging bout duration in daylight (hours)	New moon	2.85±1.29	2.61±0.97	3.25±1.59	3.52±0.98	Stage***
	Quarters	2.66±1.35	2.8±1.24	3.27±1.55	3.48±1.05	Moon $^{n.s.}$
	Full moon	2.75±1.03	2.73±0.94	3.03±1.31	3.33±1.05	Interaction $^{n.s.}$
Foraging bout duration in darkness	New moon	2.17±1.06	2.08±1.14	2.22±1.51	2.3±0.82	Stage $^{n.s.}$
	Quarters	2.08±1.03	2.3±2.77	2.15±0.85	2.41±1.02	Moon*
	Full moon	1.83±1.03	1.52±0.66	1.79±1.02	2.14±0.93	Interaction $^{n.s.}$
Flight bout duration (hours)	New moon	1.7±0.36	1.72±0.30	1.67±0.54	1.7±0.34	Stage**
	Quarters	1.95±0.40	1.87±0.34	1.63±0.22	1.81±0.47	Moon***
	Full moon	2.04±0.39	2.04±0.47	1.71±0.41	1.83±0.44	Interaction $^{n.s.}$
Flight bout duration in daylight (hours)	New moon	1.71±0.40	1.61±0.20	1.53±0.63	1.61±0.33	Stage*
	Quarters	1.75±0.47	1.63±0.28	1.46±0.26	1.62±0.35	Moon $^{n.s.}$
	Full moon	1.77±0.49	1.73±0.57	1.56±0.72	1.73±0.49	Interaction $^{n.s.}$

	Moon phase	Outward Migration	Return Migration	Stopovers	Winter	GLM
Flight bout duration in darkness	New moon	1.7±0.520	1.83±0.52	1.82±0.63	1.78±0.56	Stage*
	Quarters	2.15±0.54	2.11±0.59	1.8±0.52	2.01±0.79	Moon***
	Full moon	2.31±0.58	2.35±0.57	1.87±0.52	1.93±0.67	Interaction*
Number of flight bouts (per day)	New moon	3.75±1.08	3.93±0.93	2.79±0.77	2.07±0.59	Stage***
	Quarters	4.09±0.96	4.32±1.28	2.5±0.82	2.3±0.87	Moon***
	Full moon	4.16±10	4.69±1.02	2.82±1.03	2.36±1.14	Interaction n.s.
Number of flight bouts in daylight	New moon	2.77±0.73	2.79±0.64	2.02±0.74	1.68±0.46	Stage***
	Quarters	2.7±0.80	2.64±0.99	1.53±0.52	1.78±0.62	Moon***
	Full moon	2.33±0.79	2.19±0.66	1.72±0.60	1.72±0.71	Interaction*
Number of flight bouts in darkness	New moon	0.98±0.65	1.13±0.55	0.77±0.58	0.4±0.30	Stage***
	Quarters	1.39±0.56	1.69±0.63	0.97±0.51	0.52±0.38	Moon***
	Full moon	1.83±0.64	2.51±0.70	1.11±0.66	0.64±0.56	Interaction***
