# Supplementary material for: Do Seabirds Differ from Other Migrants in Their Travel Arrangements? On Route Strategies of Cory’s Shearwater during Its Trans-Equatorial Journey
Source: PLoS One. 2012 Nov 7;7(11):e49376. doi: 10.1371/journal.pone.0049376 (PMC3492286; doi:10.1371/journal.pone.0049376)
Supplement: Methods S1 — Effect of the loggers on the probability of return to breed on the following breeding season. (PDF) [file pone.0049376.s004.pdf]

Supporting information to manuscript: *Do Seabirds Differ from Other Migrants in their Travel Arrangements? On Route Strategies of Cory's Shearwater during its Trans-Equatorial Journey* by Maria P. Dias, José P. Granadeiro & Paulo Catry

Methods S1. Effect of the loggers on the probability of return to breed on the following breeding season. Details of the analysis to detect detrimental effects of geolocators on the probability of the birds returning to the colony to breed in the season following the deployment.

The probability of returning to breed in the year following a deployment (as opposed to not returning or returning but not breeding) was compared between birds with and without geolocator using a logistic regression. Data was also obtained from a sample of birds without loggers (n = 88) from a parallel long-term study carried out in the same colony (see [1]). The effects of the geolocator and of the year of deployment were included as potential explanatory variables in the model; the breeding success on the year of deployment was also considered, given that it is known to influence the probability of breeding in the subsequent year.

After controlling to the effects of year and breeding success, we did not find any significant effect of the geolocator ($P = 0.16$) on the probability of returned to breed during the following breeding season. The overall proportions of birds that return to breed were similar between birds with and without geolocator (83% and 74%, respectively).

1. Ramos R, Granadeiro JP, Nevoux M, Mougin J-L, Dias MP, Catry P (2012) Combined Spatio-Temporal Impacts of Climate and Longline Fisheries on the Survival of a Trans-Equatorial Marine Migrant. PLoS ONE 7: e40822.
