# Supplementary material for: Do Seabirds Differ from Other Migrants in Their Travel Arrangements? On Route Strategies of Cory’s Shearwater during Its Trans-Equatorial Journey
Source: PLoS One. 2012 Nov 7;7(11):e49376. doi: 10.1371/journal.pone.0049376 (PMC3492286; doi:10.1371/journal.pone.0049376)
Supplement: Methods S2 — Identification of the foraging bouts. (PDF) [file pone.0049376.s005.pdf]

Supporting information to manuscript: *Do Seabirds Differ from Other Migrants in their Travel Arrangements? On Route Strategies of Cory's Shearwater during its Trans-Equatorial Journey* by Maria P. Dias, José P. Granadeiro & Paulo Catry

Methods S2. Identification of the foraging bouts. Detailed procedures related with the identification of the foraging bouts using a maximum likelihood approach

The foraging bout interval criterion (or bout ending criterion – BEC; i.e., the minimum duration of an event - wet or dry - that was considered to split foraging bouts) was calculated using diveMove package [1] developed for the software R.

We first calculated the duration of all wet or dry intervals for each individual. We excluded all intervals that lasted less than 200 s, to avoid the inclusion of short-term variations in wet/dry status, which could be due to causes other than long events of foraging. The calculation of the BEC was carried out using the functions "boutinit", "bouts.mle" and "bec2" in package diveMove. The distribution of the durations of events (t) is assumed to be a mixture of two random Poisson processes: a fast and a slow one [2]. The log likelihood of all the absolute differences in event duration can be expressed as a function of p (the proportion of fast to slow events in the sampled population) and of the probabilities of an event occurring in a given process (fast or slow) per unit time (λ_f and λ_s, respectively) (see equation (3) in [3]). These parameters were calculated through an optimization procedure using appropriate initial values, which were calculated using the broken-stick method (Figure A).

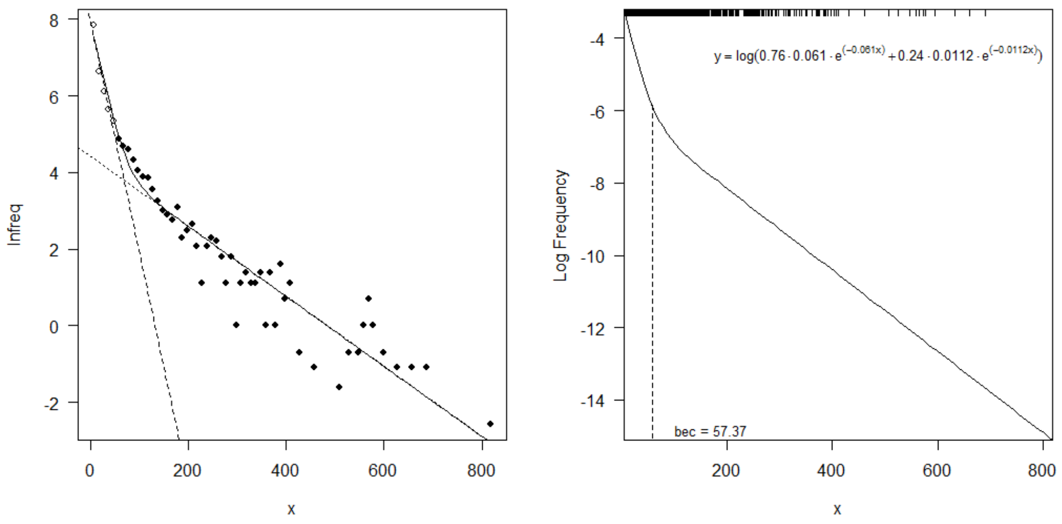

Figure A: Example of the application of the maximum-likelihood method to the calculation of the bout interval criterion (x=event duration, in minutes). Left panel: distribution of duration of events (ln-transformed frequencies; open symbols represent the fast process, and the closed symbols the slow process); Right panel: final maximum likelihood function.

The initial values obtained were then used to maximize the above-mentioned function, and then the BEC was finally calculated using the equation (4) in [3]. The values of the BEC obtained for the different individuals were averaged to obtain a final estimate of BEC (57±2 min).

References:

1. Luque SP (2007) Diving Behaviour Analysis in R. R News 7: 8–14

2. Sibly RM, Nott HMR, Fletcher DJ (1990) Splitting behaviour into bouts. Anim Behav 39: 63–69

3. Luque SP, Guinet C (2007) A maximum likelihood approach for identifying dive bouts improves accuracy, precision and objectivity. Behaviour 144: 1315–1332.
